# Robust Indoor Positioning with Smartphone by Utilizing Encoded Chirp Acoustic Signal

**DOI:** 10.3390/s24196332

**Published:** 2024-09-30

**Authors:** Bingbing Cheng, Ying Huang, Chuanyi Zou

**Affiliations:** 1 State Key Laboratory of Information Engineering in Surveying, Mapping and Remote Sensing, Wuhan University, Wuhan 430079, China; 2 School of Automobile and Information Engineering, Guangxi Eco-Engineering Vocational and Technical College, Liuzhou 545005, China; huangying800816@163.com; 3 School of Electronic Information, Wuhan University, Wuhan 430072, China; 4 School of Geodesy and Geomatics, Wuhan University, Wuhan 430079, China; chuanyizou@whu.edu.cn

**Keywords:** encoded chirp acoustic signal, TDOA, multipath and NLOS, smartphone

## Abstract

Recently, indoor positioning has been one of the hot topics in the field of navigation and positioning. Among different solutions on indoor positioning, positioning with acoustic signals has its promise due to its relatively high accuracy in the line of sight scenarios, low cost, and ease of being implemented in smartphones. In this work, a novel acoustic positioning method, called RATBILS, is proposed, in which encoded chirp acoustic signals are modulated and transmitted by different acoustic base stations. The smartphones receive the signals and perform the following three steps: (1) preprocessing; (2) time of arrival (TOA) estimation; and (3) time difference of arrival (TDOA) calculation and location estimation. In the preprocessing stage, we use band pass filters to filter out low-frequency noise from the environment. At the same time, we perform a signal decoding function in order to lock onto the positioning source. In the TOA estimation stage, we conduct both coarse and fine detection to enhance the accuracy and robustness of TOA estimation. The primary goal of coarse detection is to establish a noise range for fine detection. The main objective of fine detection is to emphasize the intensity of the first arrival diameter and resistance with multipath and non-line-of-sight (NLOS) caused by human body obstruction. In the TDOA calculation and location estimation stage, we estimate the TDOA based on the TOA estimation and then use the TDOA results for position estimation. In order to evaluate the performance of the proposed RATBILS system, two indoor field tests are carried out. The test results show that the RATBILS system achieves a positioning error of 0.23 m at 92% in region 1 of scene 1 and is superior to the traditional threshold method. The RATBILS system achieves a positioning error of 0.56 m at 92% in region 2 of scene 1 and is superior to the traditional threshold method. In scene 2, the maximum average positioning error was 1.26 m, which is better than the 3.33 m and 3.87 m of the two traditional threshold methods.

## 1. Introduction

The Global Navigation Satellite Systems (GNSS) are widely and consistently utilized for outdoor positioning and navigation services [1,2]. However, when it comes to indoor positioning, challenges arise due to signal scattering, attenuation, and the multi-path propagation effects of wireless signals. As a result, the positioning performance degrades largely indoors [3,4]. In the meantime, indoor positioning is of great significance. To name a few, it is the basis for emergency safety, crowd monitoring, precision marketing, entertainment and life, and human social needs [5,6].

Currently, methods of indoor positioning include Bluetooth, Wi-Fi, ultra wide band (UWB), inertial measurement unit (IMU), and audio. Each technology has its own characteristics. Specifically, Wi-Fi [7,8] and Bluetooth positioning with fingerprint technologies [9,10] are easy to implement and compatible with mobile devices. However, the fingerprint-based positioning method [11] requires pre-collecting a location fingerprint database, which is time-consuming and labor-intensive. UWB is able to achieve high accuracy positioning with the triangulation method [12,13]. However, the cost of the UWB module is high, while the technology has not been widely supported by current smartphones. IMUs [14,15] are frequently used in indoor pedestrian positioning systems due to their small size and low cost. However, the cumulative error limits their application in long-term positioning.

Acoustic-based indoor localization systems (AIPSs) [16,17,18,19,20] offer several advantages over radio frequency (RF)-based [21] and IMU-based positioning systems. The advantages include the following: (1) Low cost: AIPSs are generally more affordable compared with RF-based. The basic components required for acoustic localization, such as microphones and speakers, are relatively inexpensive and widely available. (2) High accuracy: AIPSs can provide high levels of accuracy in indoor environments. Acoustic waves propagate at a lower speed compared with RF signals, allowing for more precise distance measurements and localization calculations. (3) High availability: Under conditions where the human body and furniture obstruct, acoustic waves will diffract and propagate towards the microphone, which means that AIPSs can function effectively in various indoor settings without significant signal degradation. (4) Easy integration and handling: AIPSs are relatively easy to integrate into existing infrastructures. They can be easily incorporated into buildings or indoor environments without requiring extensive modifications. Furthermore, the handling and maintenance of AIPSs are typically straightforward. Due to these advantages, acoustic-based indoor localization systems have gained prominence in the field of ranging and positioning technologies alongside RF and IMU-based systems.

Based on the above property, researchers are now investigating the possibility of using acoustic signals for indoor positioning. An actual linear frequency modulation (LFM) signal was applied as the positioning source (the LFM signal is also called a chirp signal). In reference [22], the author estimated the TOA of a chirp acoustic signal. However, the author did not achieve positioning for a smartphone. In the article [23], the author uses indoor acoustic fingerprints to achieve room-level positioning and differentiation. However, the actual application is greatly affected by environmental noise because of a lack of acoustic base. In reference [24], chirp signals increasing linearly in frequency are used to code the one. A chirp signal decreasing linearly is used to code the zero. When the acoustic node information is decoded, it is considered that the smartphone and the acoustic node are in the same area. However, the system cannot obtain TDOA information. Therefore, decimeter-level positioning cannot be achieved. In the article, the author proposed a transmission scheme of time division multiple access (TDMA) plus frequency division multiple access (FDMA). However, the number of acoustic nodes in this scheme is limited because of hardware conditions. It is difficult to deploy acoustic nodes in practical applications. Meanwhile, the system only generated two codes assigned to four acoustic nodes, which can easily lead to incorrect identification of acoustic nodes. In reference [25], the author employed the PRN signal to encode the transmitted audio signal. Due to the absence of up-conversion for the PRN signal, the received signal at the receiver end is susceptible to environmental noise interference. In reference [26,27], the author uses a microphone array module to locate the target. However, the system is suitable for robot platforms and is not suitable for users in daily life. In reference [28], the author uses TDOA as a fingerprint to locate the sound source. The capacity of users is limited in such systems. At the same time, the TDOA-fingerprint method requires collecting fingerprint information in advance, which is time-consuming and laborious. In reference [29], the authors conducted a study on utilizing FDM-CDMA sound signals for indoor localization of unmanned aerial vehicles (UAVs). They employed time of arrival (TOA) information obtained through the maximum value of cross-correlation. However, it is worth noting that actual indoor environments present challenges such as multipath interference resulting from obstructions. As a result, the maximum value of the cross-correlation detection method may not be suitable for real-world scenarios. In reference [30], the authors utilized a threshold method for TOA estimation and developed a frequency division, spatial division, and time division positioning system, achieving good positioning results. However, this system has two key shortcomings: (1) The TDOA measurement method uses signals from adjacent base stations for measurement (for example, ${TDOA}_{12}$, ${TDOA}_{23}$ and ${TDOA}_{34}$). This is because the system adopts ARM architecture, resulting in a clock offset issue. (2) The system’s encoding signals are only two, allocated to multiple base stations. It is necessary to inform in advance in which area. Multiple base station identifications cannot be achieved. In reference [31], the author utilized an acoustic single base station to measure the relative displacement of mobile phones, combined with PDR information, achieving good positioning results. However, in this system, the clock of the acoustic single base station will drift over time, and the author did not analyze this issue. Furthermore, the author did not encode the signals or discuss and analyze multipath issues. In reference [32], the author utilized the semantic information of acoustic signals for indoor localization. However, in practical applications, it is susceptible to factors such as noise and human obstruction, making it impractical for real-world scenarios. In reference [33], the author proposed a combination of the normalization method and threshold method for detecting the first path. However, in practical scenarios with strong shadowing, the strength of the first path is attenuated, making it challenging to determine an appropriate threshold. In reference [34], the author proposed a positioning scheme for underground spaces utilizing acoustic signals. However, there was a lack of detailed discussion regarding the impact of multipath and potential solutions to mitigate it. To further elaborate, we categorize the above explanations. In terms of signal layer research, the authors used chirp in articles [22,30,31], which cannot simultaneously satisfy the coding of multiple base stations. Regarding ranging capabilities, in articles [28,32], effective ranging could not be achieved. In terms of system synchronization, articles [30,31] did not achieve true synchronization and did not discuss clock offset in the system. For signal TOA and TDOA estimation, in articles [33,34], the authors did not propose improved methods and continued to use the classic threshold method and cross-correlation maximum method. Regarding user capacity, in articles [25,26,27], the system is not applicable to multiple users. When multiple users transmit signals simultaneously, the system cannot locate them.

Based on the above discussions, exploring robust acoustic positioning systems still has its significance in both academia and industrial fields. In this work, a RATBILS system is developed, which only requires users to carry mobile phones for direct positioning without the need for additional auxiliary devices. Due to detrimental factors such as high levels of noise, echoes, multipath propagation, and the doppler effect in indoor acoustic channels, the quality of the transmitted signal is reduced, limiting the communication range and resulting in errors in demodulation. Traditional digital modulation techniques for wireless communication, such as amplitude, and phase modulation, are not directly suitable for our system. Additionally, it is worth noting that acoustic waves experience considerably higher attenuation (i.e., decrease in signal strength with distance) compared with electromagnetic waves of similar frequencies, resulting in low-amplitude received signals and limiting the operating range of the system. As stated in article [35], the achievement was limited to acoustic communication within a range of 0.7 m. In the fields of underwater acoustic communication [36,37,38] and wireless communication [39,40,41], chirp spread spectrum (CSS) techniques are recognized as highly efficient information transmission schemes. In CSS transmission schemes, the receiver employs matched filtering (MF) to optimize the signal-to-noise ratio (SNR), resulting in an extended communication range. Furthermore, these techniques exhibit exceptional effectiveness in dealing with low-amplitude received signals, interference, and selective fading. Based on CSS, we have developed the FDM-CSS solution, which balances positioning performance and coding effectiveness.

During the positioning process, acquiring distance information is a crucial step for smartphones. However, challenges arise in obtaining reliable distance information due to factors like indoor multipath and human obstruction. Even though we utilize the smartphone’s internal microphone for signal reception, its quality is compromised by the presence of the phone case. The signal received is inferior to that obtained by using the custom-made microphone. To enhance the reliability of distance measurement, we have devised coarse detection and fine detection techniques. During the coarse detection stage, we employ a combination of spectral subtraction and MF-backtracking to ascertain the approximate starting position of the receiving signal. During the fine detection stage, we combine the multi-threshold grouping method with normalization techniques to enhance the intensity of the first path and improve the accuracy of detection.

Acoustic indoor positioning technology leverages microphones and speakers for localization, and it possesses the following characteristics: Due to the relatively slower propagation speed of sound compared with radio frequencies, the synchronization requirements for achieving high-precision acoustic positioning are reduced. For location-based service providers, although deploying acoustic nodes indoors is necessary for acoustic indoor positioning technology, the affordability of commercial acoustic components is expected to enable cost-effective infrastructure investments while achieving sub-meter-level high-precision indoor positioning. For users, since microphones and speakers are standard features on handheld mobile devices, they can receive high-precision positioning services without any additional costs. These advantages have sparked an increasing interest among researchers in the field of acoustic indoor positioning technology. We also took these advantages into consideration and developed the RATBILS system. Specifically, we have conducted the following work:

(1) We have designed an active sensing system that allows smartphones to determine their location information without requiring any additional sensors for users.
(2) We propose a robust time-delay estimation algorithm, referred to as the coarse detection and fine detection method, which provides a reliable guarantee for accurate TDOA measurements and localization.
(3) We have designed a FIR-MF detector for detection of encoded chirp signals transmitted by different acoustic nodes.
(4) Our method is capable of adapting to adverse conditions such as human body occlusion and strong multipath interference through coarse and fine detection.
(5) Our extensive experimental results demonstrate that our proposed system exhibits accuracy and robustness for smartphone localization across two real-world scenarios.

## 2. System Overview

### 2.1. Transmission Mechanism of the Acoustic Positioning System

**Figure 1:**
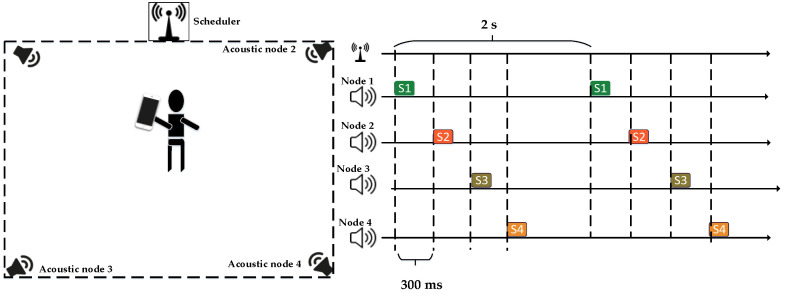
Acoustic positioning system for a smartphone.

As shown in Figure 1, we present the overall schematic diagram of the acoustic positioning system. In the localization system, multiple acoustic nodes are utilized to emit signals, and the smartphones are the receivers, where microphones are integrated. These acoustic nodes are separately placed in the room in order to achieve effective localization. In the localization system, the time of each transmitter is synchronized to the same timeline through a wireless scheduler. The wireless module in the wireless scheduler is a 433 MHz wireless module. The 433 MHz wireless module is driven through a serial port protocol. The scheduler transmits data every 2 s to synchronize all acoustic nodes. Since the speed of wireless signal propagation is 106 times that of acoustic waves, all acoustic nodes are synchronized to transmit signals on the same time axis. Our synchronization accuracy is approximately around 0.5 ms.

**Figure 2:**
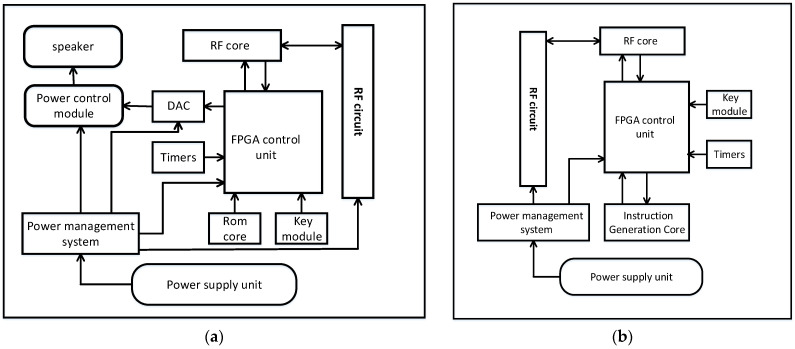
Hardware frame diagram (**a**) Acoustic node hardware architecture; (**b**) Scheduler hardware architecture.

The hardware structure of the acoustic node is shown in Figure 2a. It mainly consists of a Field-Programmable Gate Array (FPGA), a Digital-to-Analog (DA) module, an RF module, and a speaker. At the same time, three IP cores are generated inside the FPGA: ROM IP, RF core, and timer, respectively. ROM IP is used to store fixed-pointed signals. The RF core is used to interact with the RF module and also interact with the FPGA control unit. The timer provides a clock to the FPGA control unit. The DA module is controlled by a clock to convert the digital audio signal into the analog signal, which directly drives the speaker. The hardware structure of the scheduler is shown in Figure 2b. The role of the scheduler is to generate wireless trigger signals for controlling the acoustic nodes. The RF core and timer in the scheduler have the same functionality as their corresponding modules in the acoustic node. The role of the instruction generation core is to generate instructions for the FPGA control unit, and the instructions are transmitted through the RF module. The key module in both the acoustic node and the scheduler has the functionality of starting or pausing the system.

### 2.2. Design of the Acoustic Signals

Chirp signals are a widely recognized source for localization due to their robustness, excellent range resolution, and simplicity. The frequency modulation of chirp signals allows the instantaneous frequency to take on different shapes, such as linear, exponential, and quadratic. In the continuous time domain, the most commonly employed modulation is linear frequency modulation. Mathematically, a linearly frequency-modulated chirp signal is defined as follows:

(1) $$s(t)=Ae^{j(2\pi (f_{0}t+\frac{f_{c}-f_{0}}{2}t^{2}))},\;0\leq t\leq T$$

where $f_{0}$ represents the starting frequency and $f_{c}$ denotes the cut-off frequency. A stands for the amplitude of the signal, while T indicates the duration of the chirp signal. Let us define $k=f_{c}-f_{0}$. For k>0, the chirp signal is known as an up-chirp, whereas for k<0, it is referred to as a down-chirp. It is widely recognized that waveforms occupying the same frequency band but with inverse and significantly different chirp rates provide a desirable balance between frequency spectrum utilization and cross-correlation suppression. 

**Figure 3:**
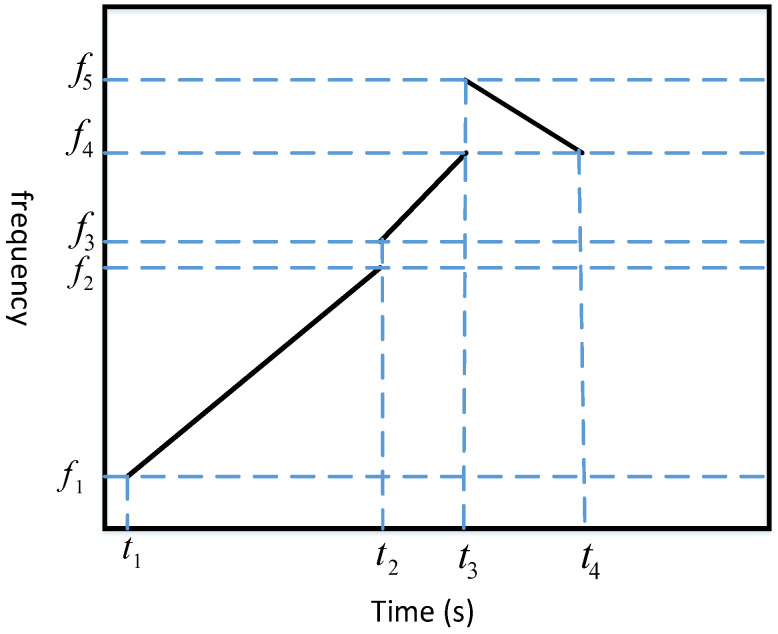
Frequency division multiplexing-chirp spread spectrum (FDM-CSS).

A classical multiple access technique is CSS multiple access technology. However, the CSS multiple access technique still has certain limitations in acoustic positioning systems. The specific reasons can be summarized as follows: (1) Interference in the same frequency band: In CSS multiple access technology, both up-chirp and down-chirp occupy the same frequency band. In practical applications, utilizing the same bandwidth will reduce the distinguishability of the signals, thereby increasing the difficulty of decoding the signals emitted by acoustic nodes. (2) Far-field and near-field effects: signals emitted by acoustic nodes located at different distances, such as far-field and near-field, are difficult to differentiate due to occupying the same bandwidth. (3) Nonlinear frequency response of acoustic nodes: The frequency response of acoustic nodes demonstrates nonlinear characteristics, indicating that as the frequency increases, the amplitude of the response decreases. This nonlinearity has the potential to affect the accuracy and reliability of decoding schemes that rely on time-frequency analysis. To address the simultaneous requirements of coding and positioning functionality, we have designed a new coding scheme called frequency division multiplexing-chirp spread spectrum (FDM-CSS), as shown in the following Figure 3.

An FDM-CSS signal frame consists of a preamble portion used for positioning and a portion used for acoustic node encoding, as shown in Figure 3. FDM-CSS has a frame structure similar to traditional CSS signals but differs from CSS technology in two aspects. Firstly, the preamble portion has a larger time-bandwidth product compared with the encoding portion, allowing the preamble to have stronger positioning capabilities. Additionally, the frequency band ranges of the preamble and encoding portions are offset. On the other hand, the encoding portion consists of multiple up-chirps and down-chirps in different frequency bands appearing alternately. The specific design concept for FDM-CSS is as follows: For each FDM-CSS signal emitted by the base station, we first determine the transmission duration T and divide T into M segments (in this paper, it is divided into three segments as shown in Figure 3, where M = 3). Subsequently, we specify the chirp signal type (down or up), as well as the start and stop frequencies for each time segment. The mathematical formula for the FDM-CSS signal specifically designed in this paper is as follows:

(2) $$s(n)=\left\{\begin{array}{c} {=s}_{p}^{1}(n)=Ae^{j(2\pi (f_{1}t+\frac{f_{2}-f_{1}}{2}t^{2}))}\;\;\;\;\;\;\;\;\;\;\;\;\;\;\;\;\;\;\;t_{1}<n<t_{2}\\ =s_{p}^{2}(n)=Ae^{j(2\pi (f_{3}t+\frac{f_{4}-f_{3}}{2}t^{2}))}\;\;\;\;\;\;\;\;\;\;\;\;\;\;\;\;\;\;\;t_{2}<n<t_{3}\\ {=s}_{p}^{3}(n)=Ae^{j(2\pi (f_{4}t+\frac{f_{5}-f_{4}}{2}t^{2}))}\;\;\;\;\;\;\;\;\;\;\;\;\;\;\;\;\;\;\;t_{3}<n<t_{4} \end{array}\right.$$

where s(n) consists of $s_{p}^{1}(n)$, $s_{p}^{2}(n)$ and $s_{p}^{3}(n)$. $s_{p}(n)$ is the FDM-CSS signal. $s_{p}^{1}(n)$ is the preamble portion of the FDM-CSS signal. $s_{p}^{2}(n)$ and $s_{p}^{3}(n)$ form the coding source of the FDM-CSS signal. Our design enables FDM-CSS to meet positioning requirement without affecting acoustic node encoding.

## 3. Robust TOA Detection for Acoustic Positioning

**Figure 4:**
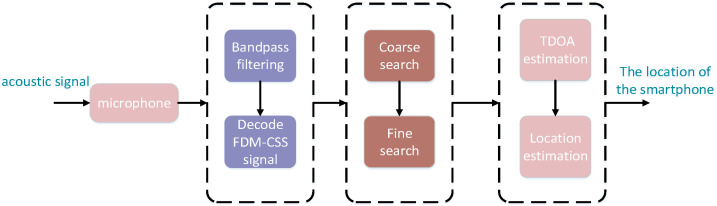
Flowchart of TDOA-based positioning.

We propose a robust time-delay estimation algorithm, referred to as the coarse detection and fine detection method, which provides a reliable guarantee for accurate TDOA measurements and localization. First, we record a segment of the acoustic signal. Due to the presence of low-frequency noise in the indoor environment, such as human voices and air conditioning sounds, it is necessary to design a high-pass filter with a passband frequency range from 14kHz to 20kHz. Next, we proceed to identify the signals emitted by the acoustic nodes, primarily utilizing the FIR-MF detector for decoding. Next, we will perform TOA estimation, with a focus on both coarse detection and fine detection. Finally, we carry out location estimation, primarily involving TDOA measurements and position calculations. As shown in Figure 4, we demonstrate the overall processing. 

### 3.1. FIR-MF Detector for Decoding FDM-CSS Signal

**Figure 5:**
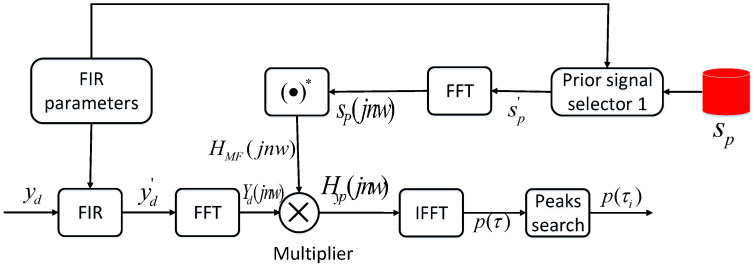
The schematic diagram of FIR-MF detector.

The distinctive feature of FDM-CSS signals is their ability to generate compressed pulses when processed through a matched filter [42,43]. It finds extensive application in radar systems due to its optimal performance in terms of signal-to-noise ratio (SNR), effectively maximizing the output SNR. By definition, a matched filter is designed specifically to match a given signal s(n). Its impulse response and frequency response are defined as follows:

(3) $$h_{MF}\left(n\right)=s^{*}(-n)$$

(4) $$H_{MF}\left(jnw\right)=S^{*}(jnw)$$

where $H_{MF}\left(jnw\right)$ is the frequency response of the matched filter and $S^{*}(jnw)$ represents the complex conjugate of the reference signal’s spectrum. The MF output is mathematically expressed as follows:

(5) $$y_{MF}\left(n\right)=y\left(n\right)⊗h_{MF}\left(n\right)$$

where $y\left(n\right)$ is the received signal, and ⊗ indicates the convolution operation. The computational complexity of this operation in the time domain corresponds to $O(\log n^{2})$; however, it could be reduced by using the equivalent in the frequency domain [44]:

(6) $$y_{MF}\left(n\right)=F^{-1}(F(x\left(n\right))\times F(h_{MF}\left(n\right)))$$

where F and $F^{-1}$ represent the fourier transform and inverse fourier transform, respectively. It is crucial to emphasize that in order to implement a fast Fourier transform (FFT) with a computational complexity reduced to O(nlogn⁡), the size of the Fourier transform should be chosen as a power of 2. Specifically, when a duplicate of the reference signal is present at the input, it will generate highly amplified autocorrelation compressed pulses at a time delay τ, resulting in prominent peaks. Conversely, it will produce lower cross-correlation peaks. So when the main FDM-CSS signal is input to a matched filter, it has the potential to generate high-amplitude compressed pulses. Based on the MF method, we have developed the FIR-MF detector to decode signals emitted from different acoustic nodes. The specific schematic diagram is shown below:

According to Figure 5, we set different parameters in the FIR parameter module to obtain different bandwidth filters in the FIR module. In this paper, based on the FDM-CSS signal configuration and number of acoustic nodes, we set a total of four FIR parameters, denoted as FIR1, FIR2, FIR3, and FIR4. In the $s_{p}$ memory, we pre-store the encoded signals of C1, C2, C3, and C4. $p(\tau_{i})$ is the maximum value of p(τ), and the approximate position of the preamble code can be determined based on $\tau_{i}$. When we obtain $p(\tau_{i})$ and $\tau_{i}$, we can decode the signal and determine the acoustic node of the positioning source.

### 3.2. Robust TOA Detection in the Multipath Inference

In practical applications, reliable TDOA measurements depend on accurate TOA estimation as a prerequisite condition. Commonly, acoustic signals are reflected by walls and windows, resulting in multiple propagation paths. As a result, signals from multiple paths continuously overlap, causing the peak obtained through cross-correlation algorithms to lag behind the peak reached by the first path. Thus, the expression for the received signal, due to the accumulation of signals from multiple paths, is as follows:

(7) $$y\left(n\right)=\sum_{i=1}^{l}a_{i}s\left(n-n_{i}\right)+Q\left(n\right)$$

where $y\left(n\right)$ represents the received signal, $s\left(n\right)$ represents the transmitted signal, $n_{i}$ represents the delay arrival time of the ith propagation path signal, Q(n) represents the noise in the environment, and there are a total of l propagation paths. To further utilize the preamble of the transmitted signal for MF, we perform band-pass filtering on $y\left(n\right)$ to remove the coded part. This yields the received signal in the form of the preamble, expressed as follows:

(8) $$y^{p}\left(n\right)=\sum_{i=1}^{l}a_{i}s_{p}^{1}\left(n-n_{i}\right)+W\left(n\right)$$

where $y^{p}\left(n\right)$ represents the received signal in the form of the preamble, $s_{p}^{1}$ represents the preamble portion of the transmitted signal, $n_{i}$ represents the delay arrival time of the ith propagation path signal, W(n) represents the noise in the environment, and there are a total of l propagation paths. Based on the prior knowledge of the transmitted signal, the MF output $Rt\left(n\right)$ between the preamble portion of the transmitted signal and $y^{p}\left(n\right)$ is as follows:

(9) $$Rt\left(n\right)=(\sum_{i=1}^{l}a_{i}s_{p}^{1}\left(n-n_{i}\right))⊗s_{p}^{1}\left(-n\right)+W\left(n\right)⊗s_{p}^{1}\left(-n\right)$$

**Figure 6:**
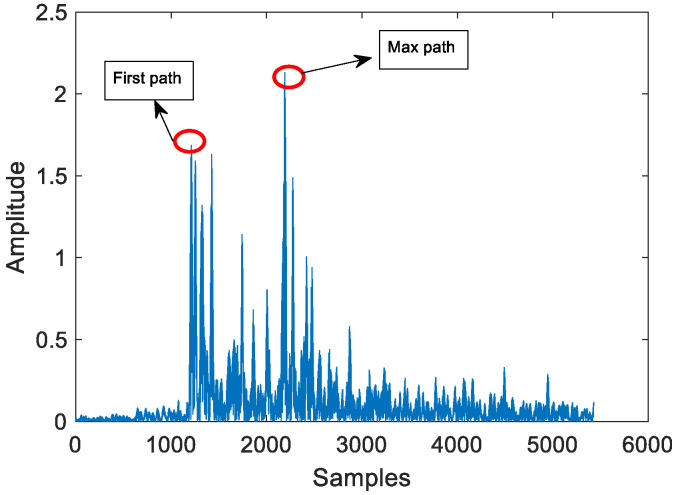
MF result within LOS condition and weak multipath.

Multipath phenomena will reduce the accuracy of TOA estimation. Figure 6 shows a schematic diagram of multipath under LOS conditions, as shown in the following figure:

To accurately locate the user’s mobile, the multipath effects have to be mitigated. In this work, we developed a method that combines coarse detection and fine detection to obtain reliable TOA estimation, ultimately resulting in robust TOA measurements.

#### 3.2.1. Coarse Detection

The purpose of coarse detection in a signal has two objectives: (1) To provide a reliable noise interval for fine detection. (2) To provide a reliable multipath interval for fine detection. Based on the two objectives, we have developed a coarse detection algorithm, which includes two steps. The spectra subtraction method and backing method are described in detail as follows: 

In step 1 of coarse search, spectral subtraction refers to the article referenced as [45].

The purpose of spectral subtraction is to reduce the impact of noise on backtracking in step 2 of coarse search. Further simplifying Formula (9), we obtain Formula (10):

(10) $$E_{R}\left(n\right)=E_{s}\left(n\right)+E_{W}\left(n\right)$$

By performing a Fourier transform on both sides of Formula (10), we have Formula (11):

(11) $$Q_{ER}\left(jnw\right)=Q_{Es}\left(jnw\right)+Q_{N}(jnw)$$

In the field of acoustics, researchers are more concerned with the magnitude spectrum information. We calculate the square of the magnitude spectrum for both sides of Formula (11). We have Formula (12):

(12) $${\left|Q_{ER}\left(jnw\right)\right|}^{2}={\left|Q_{Es}\left(jnw\right)\right|}^{2}+{\left|Q_{N}\left(jnw\right)\right|}^{2}+2Re(Q_{Es}\left(jnw\right){Q_{N}}^{*}(jnw))$$

For noise power spectral density ${\left|Q_{N}\left(jnw\right)\right|}^{2}$, we typically choose the average power spectral density of the first few frames of the received signal instead. In order to estimate ${\left|Q_{Es}\left(jnw\right)\right|}^{2}$, we assume that ${\left|Q_{N}\left(jnw\right)\right|}^{2}+2Re\left(Q_{Es}\left(jnw\right){Q_{N}}^{*}\left(jnw\right)\right)\approx \alpha {\left|Q_{N}\left(jnw\right)\right|}^{2}$, then we have the following formula:

(13) $${\left|Q_{ER}\left(jnw\right)\right|}^{2}\approx {\left|Q_{Es}\left(jnw\right)\right|}^{2}+\alpha {\left|Q_{N}\left(jnw\right)\right|}^{2}\;\;\;\;\;\alpha \geq 1$$

Based on Formula (13), we let $D\left(k\right)={\left|Q_{N}\left(jnw\right)\right|}^{2}$ and have the following equation:

(14) $${\left|Q_{Es}\left(jnw\right)\right|}^{2}\approx \left\{\begin{array}{c} {\left|Q_{ER}\left(jnw\right)\right|}^{2}-\alpha D\left(k\right)\;\;\;\;\;{\left|Q_{ER}\left(jnw\right)\right|}^{2}\geq \alpha D(k)\\ \beta D\left(k\right)\;\;\;\;\;\;\;\;\;\;\;\;\;\;\;\;\;{\left|Q_{ER}\left(jnw\right)\right|}^{2}<\alpha D(k) \end{array}\right.$$

**Figure 7:**
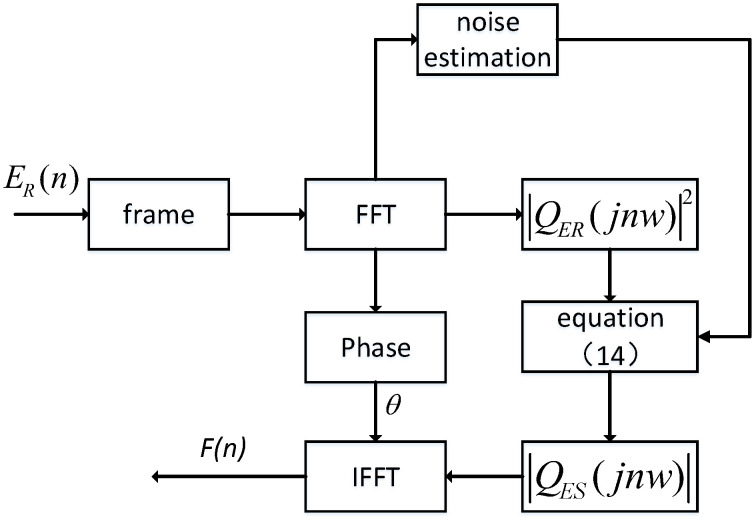
Spectral subtraction flowchart.

In this article, α is set to 2, and β is set to 0.01. Next, we convert the frequency domain signal into the time domain signal, with the specific formula as follows:

(15) $$F\left(n\right)=ifft(\left|Q_{Es}\left(jnw\right)\right|e^{j\theta })$$

where ifft represents the Inverse Fast Fourier Transform, $\left|Q_{Es}\left(jnw\right)\right|$ represents the frequency domain magnitude, θ represents the phase of $Q_{ER}\left(jnw\right)$, and $F\left(n\right)$ represents the time domain signal. Figure 7 is the overall flowchart of spectral subtraction, as shown in the following figure:

In step 2 of coarse search, we use the backtracking method to obtain the starting time of the coarse extraction signal. In the backtracking method, we first identify the discrete time corresponding to the maximum peak value and then provide multipath interval and noise interval for fine detection through backtracking. The backtracking process is as follows:

In Algorithm 1, F(n) is the absolute value result after spectral subtraction. CR represents the starting time of the coarse extraction signal. The parameter ty represents the scale factor for backtracking. In both experimental scenes of this paper, ty is set to 94. In both experimental scenes of this paper, WR is the span factor of the backtracking algorithm, which is set to 400 in both experimental scenarios of this article.
**Algorithm 1**. Backtracking Input: F(n)
 Output: CR
 1: HD=max(F(n)). (HD is the maximum value of F(n)) 2: Ba=argmax(F(n)). (Ba is the discrete time corresponding to the maximum value of F(n).) 3: $F\left(Ba+1:end\right)=0$. 4: cj=1
 5: Backtracking search:            for h = 1:ty
$CNT=\mathrm{find}\left(F\left(n\right)>\left(1-(h-1\right)*0.01\right)*HD)$CT(cj)=CNT(end)            if $CT\left(cj\right)>20$
$F\left(CT\left(cj\right)-20:end\right)=0$            endcj=cj+1            endCT=CT(end:−1:1).$CR=CT\left(1\right)-WR$ 6: Return: CR


**Figure 8:**
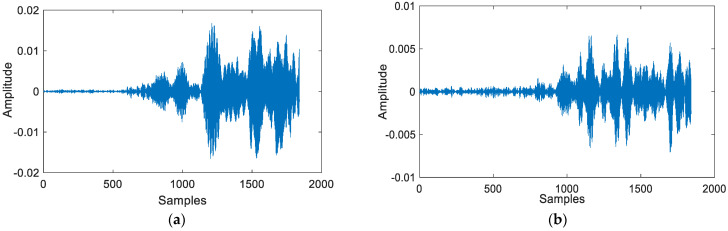
Coarse detection result in different conditions (**a**) Coarse detection result within LOS condition; (**b**) Coarse detection result within NLOS condition.

**Figure 9:**
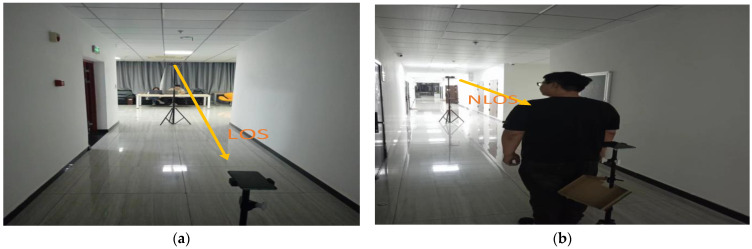
Experiment in different conditions (**a**) Experiment within LOS condition; (**b**) Experiment within NLOS condition.

According to Figure 8, it is evident that under NLOS conditions, the signal experiences a more pronounced attenuation. Furthermore, the signal near the first path is nearly completely overshadowed by noise. Therefore, we need to conduct a fine search to improve the accuracy of TOA measurements. Figure 9 shows experiments under different conditions, corresponding to the signal in Figure 8

#### 3.2.2. Fine Search

**Figure 10:**
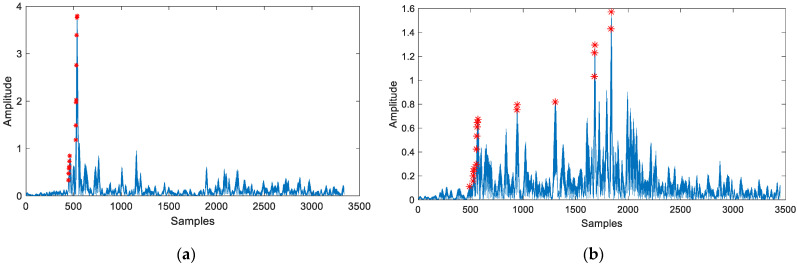
Multiple threshold extracting results in different conditions (**a**) Multiple threshold extracting results within LOS condition (red asterisk represents $R_{i}\left(Tq(n)\right)$); (**b**) Multiple threshold extracting results within NLOS condition (red asterisk represents $R_{i}\left(Tq(n)\right)$).

In Algorithm 1 of coarse detection, we can obtain the coarse detection signal through the output CR. Let the coarse detection signal be $R\left(\tau \right)=Rt\left(CR:CR+3400\right)$. At the same time, we assume $R_{i}$ is the result of taking the absolute value of $R\left(\tau \right)$. Due to the effects of multipath and human body blocking, the strength of the first path of the signal is not always the maximum value. Therefore, the classical method for detecting the TOA is the threshold method. However, a single threshold method is not applicable to all positions in the same scene. Therefore, this paper proposes improvements based on the threshold method. The classical threshold method for detecting the *TOA* is as follows:

(16) $$TOA=argmin\left(\left|R\left(\tau \right)\right|\geq THR\cdot \max(\left|R\left(\tau \right)\right|)\right)$$

where $R\left(\tau \right)$ is the result of MF. THR is the threshold. In the classical threshold method, the threshold is a fixed value. Based on the classical threshold method, we divide the fine search method into the following two steps:

Step 1: Multiple threshold grouping. In article [44], the author proposes that the use of a multi-threshold method can yield peaks at different time indices and categorize these peaks into different groups. However, the author incorporates all potential first paths of each group into the localization process, which increases the complexity of localization. Additionally, the article also points out that if the signal is strongly influenced by reverberation, the number of groups will be greater. In article [46], the author utilizes the formula $\pm \frac{F_{S}}{B}$ to reflect the width of the main lobe after MF ($F_{S}$ represents the sampling rate, *B* represents the bandwidth of the signal), which reflects the aggregation of MF output results in ideal conditions. Combining the two facts, we have developed a multi-threshold grouping method. First, we set a range for the threshold THR in Equation (16), THR∈[pr:δ:1]. By gradually increasing THR from pr to 1 with a fixed step size δ, we can obtain a time series called $Tq\left(n\right)$, where n = 1, 2, …, $\frac{1-pr}{\delta }+1$. In other words, $Tq\left(n\right)$ is a discrete time series related to TOA estimation. Due to the effects of multipath and human body obstruction, the received signal exhibits certain clustering characteristics after MF processing, as shown in Figure 10. If peaks gather together, we consider them to belong to the same group. Subsequently, as shown in Equation (17), we can derive a time difference sequence $IV\left(n\right)$, which captures the time interval between consecutive time indices of $Tq\left(n\right)$.

(17) $$IV\left(n\right)=Tq\left(n+1\right)-Tq\left(n\right)\;\;\;\;\;\;\;\;\;\;\;n=1,\;2\ldots \frac{1-pr}{\delta }$$

From the analysis of Figure 10, it is evident that $Tq\left(n\right)$ can be divided into several groups. The fundamental reason for the existence of multiple clusters is due to multipath and human body blockage effects. The specific, detailed process of step 1 is shown in Algorithm 2.
**Algorithm 2**. Multiple Threshold Grouping Input: $R_{i}$. Output: GD. 1: Set threshold THR (THR∈[pr:δ:1])). 2: Set δ=0.01. 3: $\rho =max(R_{i})$.  4: Find $Tq\left(n\right)=argmin\left(R_{i}\geq THR\left(n\right)*\rho \right)$, n = 1, 2, ..., $\frac{1-pr}{\delta }+1$. 5: Group decision:
      (1)Set variable *GD* = 1, *GD* represents the number of the group.      (2)for *n* = 1:$\frac{1-pr}{\delta }$$IV\left(n\right)=Tq\left(n+1\right)-Tq\left(n\right);$             end for             for *n* = 1:$\;\frac{1-pr}{\delta }-1$
             if $IV\left(n\right)\;$< TB
${index}_{i}\left(n\right)\;Belongs\;to\;group\;GD;$                    else*GD* = *GD* + 1;$Tq\left(n+1\right)\;Belongs\;to\;groupGD$;                    end if      end for

In Algorithm 2, $R_{i}$ is the coarse extraction result obtained based on CR and $Rt\left(n\right)$. *GD* is the number of groups. TB represents the basis for grouping. We further define TB as follows:

(18) $$TB=\partial \times \frac{F_{S}}{B}$$

where $F_{S}$ represents the sampling rate and is set to 48 kHz in this paper. B represents the bandwidth of the signal, which is set to 3 kHz in this paper. ∂ represents the adjustment factor and is set to 1.5, which reflects the maximum aggregation degree of each group. When ∂=1.5 and TB = 24, it meets the actual grouping requirements. When ∂ is smaller, there will be more groups, which will increase the computational complexity. When ∂ is larger, there will be fewer groups, which will reduce the accuracy of TOA estimation. In the two scenarios of this paper, the pr is set to 0.06. If the pr is set too high, it may miss the first path. If the pr is set too low, such as 0.02, it may introduce noise. The experiment shows that the 0.06 is good.

In Figure 10, the multiple threshold method can extract the time series *Tq*(*n*), and the red asterisk in the Figure 10 is represented by $R_{i}\left(Tq(n)\right)$. It is obvious that under NLOS condition, the red asterisk can be divided into more groups. Meanwhile, we can also clearly see that the red asterisks are more concentrated in LOS condition. For Tq(n), the maximum value of n is $\frac{1-pr}{\delta }+1$, and in this article, the maximum value of n is set to 95. So there are a total of 95 red asterisks. The horizontal axis of the red asterisk is Tq(n). The vertical axis of the red asterisk is $R_{i}\left(Tq(n)\right)$.

**Figure 11:**
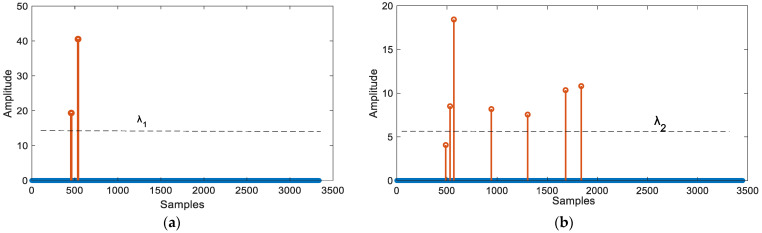
Normalized results in different conditions (**a**) Normalized results within LOS condition; (**b**) Normalized results within NLOS condition.

Step 2: Normalization. In both article [33] and article [47], the authors have proposed the concept of normalization. However, their normalization was not performed based on the coarse extraction. When the noise segment length is very large, the first path intensity cannot be highlighted. We follow the principle of normalization to normalize the grouped sequences. By performing normalization, the SNR of each sequence group can be reflected. We assume there are k sequences in the jth group, with the first sequence corresponding to time ${tm}_{1}^{j}$ and the kth sequence corresponding to time ${tm}_{k}^{j}$. Then we have the following definitions:

(19) $$NR(j)=\frac{R_{i}({tm}_{k}^{j})}{mean(R_{i}(1:{tm}_{1}^{j}))}$$

where $mean(R_{i}(1:{tm}_{1}^{j}))$ is the average of $R_{i}$ at 1$\leq \tau \leq {tm}_{1}^{j}$ and NR(j) represents the ratio of the maximum value of the jth group to the noise level. In line-of-sight (LOS) conditions, if the estimated TOA is located in the jth group, the maximum value of the jth group should be much larger than the noise level. However, due to human blockage and the influence of strong multipaths, the NR(j) of the jth group where the first path is located is reduced. According to Figure 11, we can observe that the first path is enhanced, regardless of whether it is under LOS or NLOS conditions. However, we still need to set an appropriate threshold to detect the first path. So the threshold parameter $\lambda_{i}(i=1,2)$ is set based on the threshold estimation experiment.

(20) $$\;TOA=\left\{\begin{array}{c} argmax\left(NR>\lambda_{1}\times \max\left(NR\right)\right)\;\;\;\;\;GD\leq ly\;\\ {argmax(NR>\lambda }_{2}\times max(NR))\;\;\;\;GD>ly \end{array}\right.$$

where *GD* is the number of the group and ly is the upper limit for grouping under LOS conditions. We can determine ly through the experiment.

## 4. Localization Algorithm and Error Evaluation

### 4.1. Robust TDOA Measurement

The benefits of the TDOA-based positioning system have been described previously. For *n* acoustic nodes, we can obtain *n* TOAs. So we can obtain *n −* 1 TDOAs by the following formula:

(21) $${TDOA}_{j}={TOA}_{j+1}{-TOA}_{1}-0.3s\times j\;\;j=1,2,3\ldots n-1$$

where ${TOA}_{1}$ is the TOA corresponding to the signal transmitted by acoustic node 1. ${TOA}_{j+1}$ is the TOA corresponding to the signal transmitted by acoustic node j+1. ${TDOA}_{j}$ is the TDOA corresponding to the signals transmitted by acoustic node 1 and acoustic node j+1.

### 4.2. TDOA-Based Localization Algorithm

In our research, we conducted two experiments for static positioning to comprehensively assess the proposed algorithm’s performance. In the static positioning experiment, we utilized the localization error (LE), which is calculated based on Formula (22), as a metric to evaluate the accuracy of positioning.

(22) $$LE=\sqrt{{\left(x-x^{’}\right)}^{2}+{\left(y-y^{’}\right)}^{2}}$$

where LE is the positioning error, (x, y) is the true position, and ($x^{’}$, $y^{’}$) is the estimation. The accuracy of positioning indirectly indicates the robustness of the TDOA detection algorithm. Although the least squares method provides an optimal solution, it ignores the influence of noise and lacks high accuracy in practical applications. Therefore, we use the maximum likelihood (ML) [48] algorithm as a localization algorithm. Without loss of generality, let us assume there are *M* + 1 acoustic nodes, denoted as $S_{1}\ldots ,S_{M+1}$. We will select $S_{1}$ as the reference sensor. The received signals in the reference and other sensors are employed to extract *M* TDOA measurements by local processing. TDOA measurement can be easily converted to RDOA measurement given the signal propagation speed. Then we have the following formula:

(23) $${RDOA}_{i1}=\left\|U-S_{i}\right\|-\left\|U-S_{1}\right\|$$

where U is the position of the smartphone and $S_{i}$ is the position of the acoustic node i. Owing to measurement noise, the observed RDOA is represented by ${RDOA}_{i1}^{*}={RDOA}_{i1}+n_{i1}$. Collecting all RDOA measurements yields in matrix form:

(24) $${RDOA}^{*}=RDOA+n$$

where $RDOA={\left[{RDOA}_{1},\ldots {,RDOA}_{m}\right]}^{T},$ and ${RDOA}^{*}$ and n are defined similarly. The noise matrix n is defined as a zero-mean Gaussian distributed matrix with covariance Q. The ML estimator of U can be expressed as follows:

(25) $$U_{ML}=argmin{\left({RDOA}^{*}-HD^{*}\right)}^{T}Q^{-1}({RDOA}^{*}-HD^{*})$$

(26) $$D^{*}={\left[\left\|U-S_{1}\right\|,\ldots ,\left\|U-S_{M+1}\right\|\right]}^{T}$$

(27) $$H=[-1_{M}E_{M}]$$

where $1_{M}$ and $E_{M}$ represent column matrix of all ones with length of M and identity matrix of size M×M, respectively. The solution corresponding to Equation (25) can be obtained by using the Gauss–Newton method [49] to solve the ML problem. One of the advantages of using the ML algorithm is its ability to easily detect abnormal localization results, making it sensitive to measurement noise.

## 5. Experiment

To evaluate the performance of the proposed TDOA detection method, we conducted experiments in two different typical indoor environments. Our experiments had three main objectives: (1) to determine the threshold parameters of the proposed signal detection algorithm; (2) to compare the performance of the proposed signal detection algorithm with the single-threshold method in indoor environments with multipath and NLOS propagation; (3) to highlight the robustness of the proposed algorithm compared with the traditional threshold method. In summary, through these experiments, our aim is to verify the robustness and practicality of the proposed algorithm in indoor environments.

### 5.1. Basic Parameters of the Experiment

In the experiment, four acoustic nodes were designed, and each node was equipped with a different encoding signal. As shown in the previous Figure 3, we need to pre-set parameters such as $f_{1}$, $f_{2}$, $f_{3},$ and $f_{4}$, the frequency parameter design of the FDM-CSS signal is shown in Table 1. In Table 2, We define $\rho_{t_{1}}=t_{2}-t_{1}$, $\rho_{t_{2}}=t_{3}-t_{2}$, and $\rho_{t_{3}}=t_{4}-t_{3}$. Table 1 below shows the frequency parameters of FDM-CSS signals. Table 2 below shows the time parameters of FDM-CSS signals.

**Figure 12:**
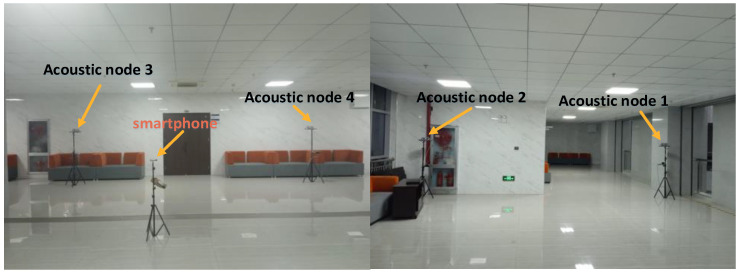
Experiment scene 2 diagram.

In two experimental scenarios, the acoustic node deployment shape is rectangular. In scenario 1, the coordinates of the four acoustic nodes are (0.0 m, 0.0 m, 2.13 m), (0.0 m, 4.8.m, 2.13 m), (4.8 m, 0.0 m, 2.13 m), and (4.8 m, 4.8 m, 2.13 m). In scenario 1, the height of the smartphone is 1.13 m. In scenario 2, the coordinates of the four acoustic nodes are (0.0 m, 0.0 m, 2.13 m), (0.0 m, 6.4 m, 2.13 m), (7.9 m, 0.0 m, 2.13 m), and (7.9 m, 6.4 m, 2.13 m). In scenario 2, the height of the smartphone is 1.13 m. The deployment diagram of the actual scenario 2 is shown in Figure 12.

**Figure 13:**
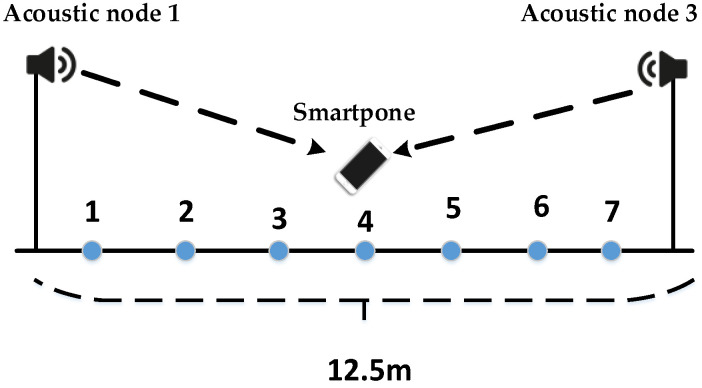
Threshold estimation experiment (corridor scene).

According to the description of the fine search algorithm, we need to determine three thresholds *(*ETP=ly*,* $LER=\lambda_{1},$ *and* $ER=\lambda_{2}$). To determine a reliable threshold ETP, we conducted signal group detection tests under LOS conditions. We choose to deploy acoustic node 1 and acoustic node 3 in a corridor environment, as shown in Figure 13.

**Figure 14:**
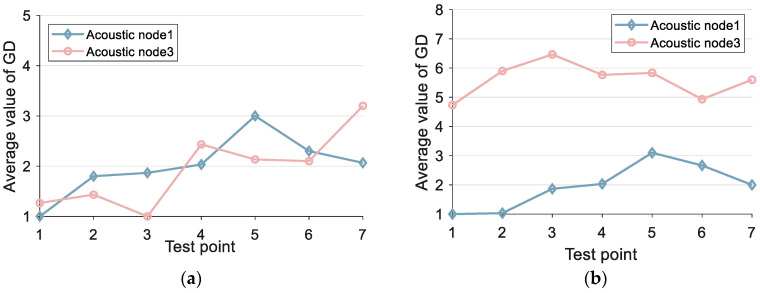
Group experiments under line-of-sight conditions and non-line-of-sight conditions: (**a**) acoustic nodes are within LOS condition; (**b**) acoustic node 1 is under LOS condition, and acoustic node 3 is under NLOS condition.

Between the two acoustic nodes, we select seven test points to collect data. We can obtain TDOA measurement and signal grouping information at each test point. At different test points, the grouping information is shown in Figure 14:

In Figure 14a, the smartphone is placed on a stand without any obstructions. In Figure 14b, the experimenter holds the smartphone, with acoustic node 3 in NLOS condition and acoustic node 1 in LOS condition. Although acoustic node 1 in both Figure 14a,b is in the LOS, Figure 14b involves a person holding the phone, and it is not under the same time conditions, which results in different experimental samples. Therefore, the group averages differ. As for the definition of ETP, we defined it in the paper as ETP=ly. ETP can be used to distinguish the state of the signal, LOS or NLOS state. We can refer to the explanation in Equation (20). In Figure 14a, we calculated the average value, and the GD is less than or equal to 3. In Figure 14b, we calculated the average grouping, and under LOS conditions, most of the sample groups are less than or equal to 3. In the NLOS condition, the majority of groups are greater than 3. Additionally, even if a small portion of LOS samples are classified as NLOS, it will not have a significant impact on the actual TOA estimation because the normalization threshold in the NLOS state is smaller. The more groups there are, the smaller the required normalization threshold. In summary, there is no contradiction. So we will set ETP to 3 based on practical applications. After determining ETP, we determine LER. We found that setting different thresholds for LER (between 0.1 and 0.4) did not result in significant changes in the LOS condition.

From Table 3, we can observe that when LER is greater than or equal to 0.2, the range measurement error tends to stabilize. In practical applications, we set LER = 0.3. After determining LER, we determine ER. By setting different values for ER and comparing the ranging accuracy, we can determine the optimal value for ER. 

**Figure 15:**
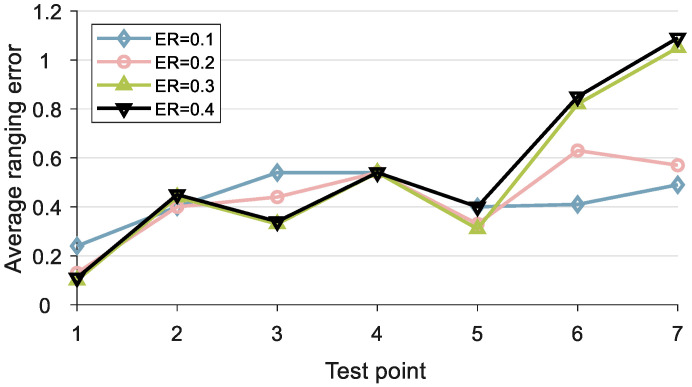
Ranging error in different ER.

According to Figure 15, we can observe that the RDOA measurement error is relatively small when the ER is set to 0.1 or 0.2. At the same time, we also found that when ER is set to 0.3 or 0.4, the measurement error of RDOA from test point 1 to test point 5 is relatively small, while the measurement error of RDOA is particularly large at test points 6 and 7. To ensure the robustness of RDOA measurements, we set the ER to 0.2 in the experiments.

### 5.2. Experimental Results and Analysis

#### 5.2.1. Performance Comparison of Different TDOA Detection Methods

To highlight the robustness of the TDOA detection algorithm in this paper, we present the TDOA measurement results for scenario 1. We compare the algorithm proposed in this paper with three traditional TDOA detection methods. Assuming the speed of sound is 340 m/s, a TDOA measurement error of 1 ms corresponds to a ranging error of 0.34 m. It is noteworthy that TDOA measurement can be easily converted to range difference of arrival (RDOA) measurement given the signal propagation speed. Therefore, TDOA and RDOA are used interchangeably throughout this paper.

**Figure 16:**
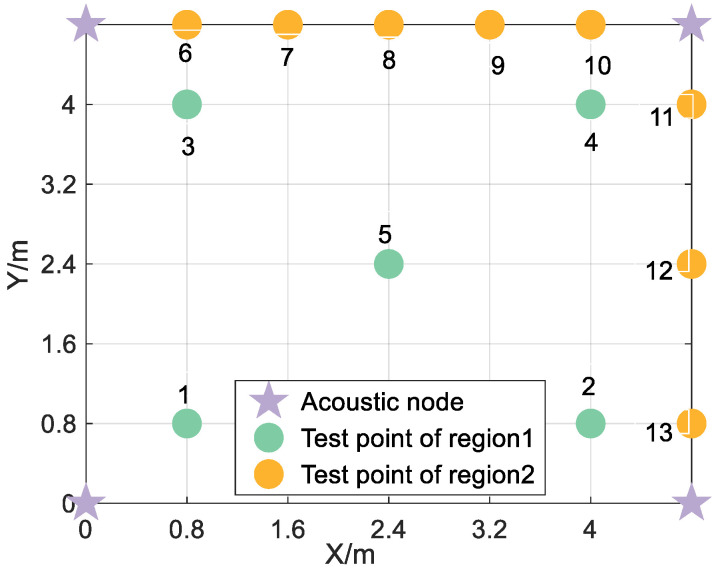
Distribution of acoustic nodes and test points in experiment scene 1.

Figure 16 corresponds to the schematic diagram of scene 1. In scene 1, we performed data collection at a total of 13 test points. These 13 testing points were further categorized into two distinct regions, namely region 1 and region 2. It is evident that the acoustic signal in region 2 is significantly affected by more pronounced obstruction and multipath effects, primarily due to the closer proximity of the testing point to the wall. We collected data 50 times at each test point, and there were human occlusion conditions during each data collection process. To compare the accuracy of different TDOA detection algorithms, we first performed RDOA measurements on the test points in region 1 of scene 1. The average RDOA measurement error was then used to evaluate the performance of different detection algorithms.

**Figure 17:**
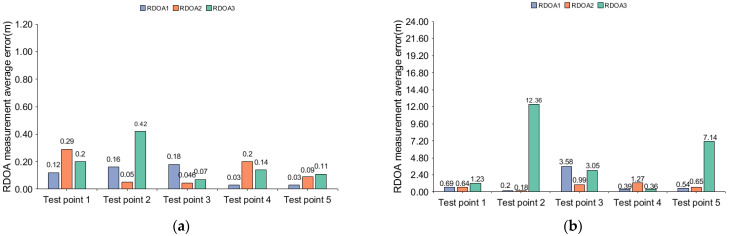
Average measurement error of RDOA in region 1 of scenario 1: (**a**) RDOA measurement average error in region 1 (using prosed method); (**b**) RDOA measurement average error in region 1 (using MF-max method).

**Figure 18:**
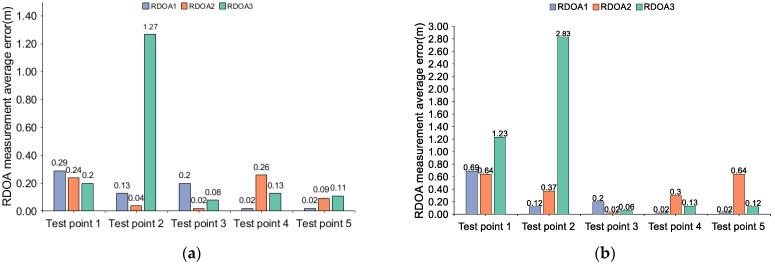
Average measurement error of RDOA in region 1 of scene 1: (**a**) RDOA measurement average error in region 1 (using MF-0.2 method); (**b**) RDOA measurement average error in region 1 (using MF-0.3 method).

**Figure 19:**
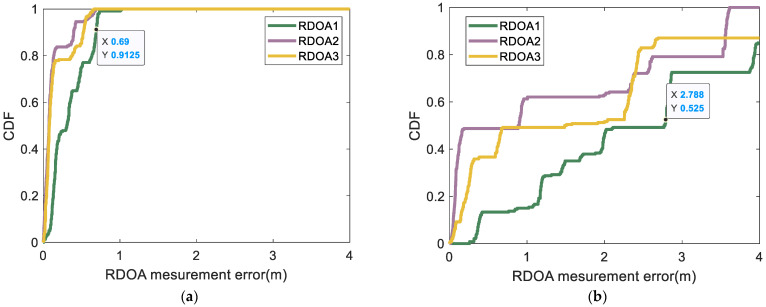
CDF of the RDOA measurement errors in region 2 of scene 1: (**a**) CDF of the RDOA measurement errors in region 2 (using proposed method); (**b**) CDF of the RDOA measurement errors in region 2 (using MF-max method).

**Figure 20:**
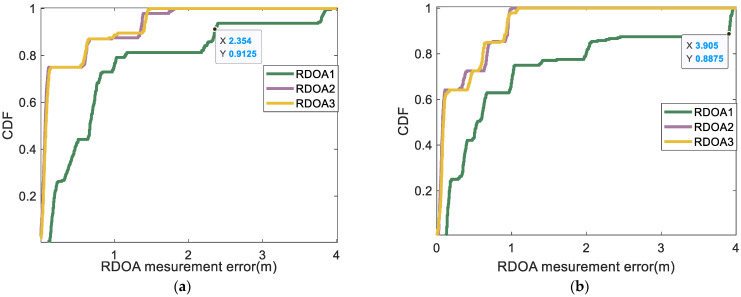
CDF of the RDOA measurement errors in region 2 of scene 1: (**a**) CDF of the RDOA measurement errors in region 2 (using MF-0.2 method); (**b**) CDF of the RDOA measurement errors in region 2 (using MF-0.3 method).

According to Figure 17 and Figure 18, the statistical results show that in region 1 of scene 1, the maximum measurement errors of the traditional threshold-based method are 1.27 m and 2.83 m, respectively, while the maximum error of the direct matching maximum value method reaches 12.36 m. However, when using the algorithm proposed in this paper, the errors are all below 0.5 m. In region 1 of scene 1, all the test points are located within the coverage of the acoustic nodes. The acoustic signals are mainly influenced by factors such as multipath and human obstruction, leading to significant ranging errors when using traditional algorithms. In region 1 of scene 1, if the ranging errors for all three RDOA measurements are within 1 m, there will be no abnormal positioning results. On the other hand, when the ranging errors are all less than 0.5 m, we consider it excellent ranging results. However, if all ranging errors exceed 1 m, there may be abnormal positioning results. Furthermore, the ranging error in this study is also dependent on the length and bandwidth of the source signal (i.e., time-bandwidth product). A larger time-bandwidth product corresponds to higher ranging accuracy. In the FDM-CSS signal, we select the signal with the maximum time-bandwidth product as the positioning source (i.e., the signal corresponding to the time interval $\rho_{t_{1}}$). This statistical result validates the robustness of the proposed algorithm. The RDOA measurement error mainly originates from factors such as multipath and non-line-of-sight conditions. Traditional algorithms lack the ability to accurately detect changes in the intensity of the first path, leading to considerable discrepancies in RDOA measurement errors across various time intervals and test points. On the other hand, the algorithm proposed in this paper achieves an error below 0.5 m for all test points in region 1 of scene 1, which further confirms its effectiveness in enhancing the intensity of the first path. To further illustrate the robustness of our algorithm, we conducted experiments at different test points in region 2 of scene 1 and provided a CDF (Cumulative Distribution Function) statistical graph:

According to Figure 19 and Figure 20, the CDF statistical results indicate that the proposed algorithm yields RDOA measurement errors predominantly within 0.7 m at the 92% percentile. The MF-0.2 method closely follows, exhibiting RDOA measurement errors within 2.3 m at the 92% percentile. Meanwhile, the employment of the MF-0.3 method results in RDOA measurement errors within 3.9 m at the 89% percentile. Notably, the largest RDOA measurement errors occur when utilizing the MF-max method, with errors within 4 m at the 80% percentile. The algorithm proposed in this article involves coarse detection and fine detection, specifically designed to address multipath and human occlusion with a high degree of precision. In region 2 of scene 1, all test points are located near the wall. Consequently, the conventional algorithm’s measurement error in RDOA would increase. Furthermore, the absorption of sound wave energy by the wall can also impact the accuracy and stability of the detection process. Additionally, factors such as phone cases and human obstructions can diminish the strength of the first path, posing challenges for reliable first-path detection when using traditional algorithms. Based on the CDF statistical results, it is evident that the algorithm proposed in this paper performs comparably well in terms of accuracy and robustness when compared with traditional algorithms, specifically in region 2 of scene 1. The utilization of the proposed algorithm ensures a harmonious trade-off between accuracy and stability in all RDOA measurements, thereby substantiating the suitability of the proposed approach. Although the algorithm proposed in this article may lead to RDOA measurement errors of up to 1 m, we believe that it could be attributed to a lower ratio between the strength of the first path and the noise intensity during the normalization stage, resulting in occasional missed detections of the first path. However, reliable positioning results can still be achieved by measuring multiple different RDOAs. Subsequently, in the following section, we will provide a detailed explanation of this phenomenon through quantifiable indicators related to the positioning result.

#### 5.2.2. Performance Analysis of Localization under Different Detection Methods

In the positioning experiment, we employed a classical ML algorithm for two specific reasons. Firstly, when dealing with RDOA measurements that exhibit significant errors, the resulting positioning values tend to approach infinity. This characteristic provides a basis for evaluating the proposed algorithm in this paper. Secondly, the ML algorithm offers higher accuracy compared with other variations of the least squares method. Since the experimental scenario in this paper is relatively small, the initial values have minimal impact on the accuracy of ML positioning. So we choose the center point of the region as the initial value for the ML algorithm. The primary objective of the positioning experiment is to assess the robustness of the TDOA detection algorithm proposed in this paper. Thus, the application of the ML positioning algorithm is more suitable. In this paper, if there are more than 8 abnormal positioning results, we consider the average positioning error to be infinite. If the number of abnormal positions is less than 8, we will exclude the abnormal positioning results and then calculate the average positioning error. The positioning outcomes for scenario 1 can be observed in Table 4.

**Figure 21:**
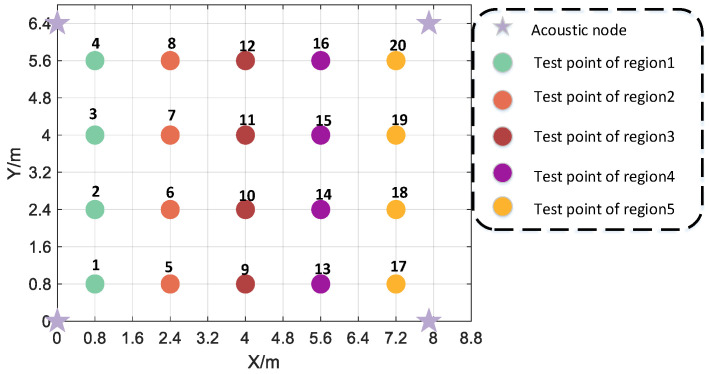
Distribution of acoustic nodes and test points in experiment scene 2.

According to Table 4, the statistical results indicate that the proposed algorithm exhibits positioning errors in region 1 and region 2 of 0.23 m and 0.56 m at the 92% percentile, respectively. By applying the threshold method, the maximum positioning errors in region 1 and region 2 increase to 1.31 m and 2.07 m, respectively. However, it should be noted that the MF-max detection method produces abnormal results, with positioning outcomes approaching infinity in certain indicators. Nevertheless, the proposed TDOA detection algorithm in this paper demonstrates better performance than traditional algorithms in various positioning indicators, thanks to its utilization of both coarse and fine detection techniques. Consequently, when employing the ML positioning algorithm, all positioning indicators surpass those of conventional algorithms. In scene 1, the positioning indicators in region 1 outperform those in region 2. This difference can be attributed to the fact that all test points in region 2 are situated near walls, resulting in more severe multipath and non-line-of-sight interference for the signals collected at these points. Furthermore, due to the ML positioning algorithm’s sensitivity to large-ranging errors, significant errors can cause the positioning results to approach infinity. These observations provide practical evidence supporting the robustness of the proposed TDOA detection algorithm. The experimental results from scene 1 further confirm the algorithm’s reliability. To further validate its effectiveness, positioning experiments were conducted at 20 test points in scene 2, as depicted in Figure 21. For each test point, 50 valid audio data samples were collected, and the average positioning results were calculated and listed in Table 5. The outcomes in Table 5 provide additional evidence supporting the algorithm’s robustness, as described in this paper. It is anticipated that these findings will hold true in other smaller-scale scenarios. However, notable positioning errors still persist in scene 2. For instance, at test point 3, the proposed algorithm yielded a positioning error of 1.26 m. This can be attributed to strong noise interference during certain moments, which makes the detection of the first path more challenging.

## 6. Conclusions

In this article, we have designed a robust acoustic localization system. The encoding scheme we designed is simple, and decoding only requires basic filtering and matching operations. The frequency used in this study is much higher than the frequency of environmental noise. However, the accuracy and stability of TDOA measurements are influenced by multipath effects and human obstruction, and the traditional threshold method cannot provide robust TDOA measurements. To address this issue, we have adopted a combined coarse detection and fine detection method to improve the robustness and reliability of the system. The proposed method significantly enhances the robustness of TDOA detection compared with the traditional threshold method. Additionally, we conducted static positioning experiments in two scenes, and the results demonstrate that the proposed method outperforms two traditional threshold methods in terms of positioning accuracy and robustness. However, there are still some issues that need to be addressed in future research:

(1) Large-scene smartphone positioning: Our acoustic nodes are easy to deploy, and we have the ability to achieve positioning in large indoor spaces. However, there is a problem of the near–far effect in large indoor spaces. We plan to overcome this problem by increasing the length of the localization source signal and using the dereverberation processing.
(2) Dynamic positioning: An acoustic signal is susceptible to doppler effects. This issue is something we need to address in the future. We plan to choose methods in the field of communication, such as carrier frequency offset compensation.
(3) Switching between dynamic positioning and static positioning: When performing the positioning function, the user may be in a stationary state or in a moving state. In moving state, we can use the extended Kalman filter to improve positioning accuracy. We plan to use TOA information to detect movement distance and determine whether it is stationary.
(4) Adaptive extraction of valid acoustic data segments: this article does not study the adaptive extraction method. However, signals from acoustic nodes can be encoded and decoded, which provides the possibility for adaptive extraction.

## Figures and Tables

**Table 1:** FDM-CSS signal parameters of different acoustic nodes.

Acoustic Node	$\left(f_{1}f_{2}\right)$	$\left(f_{3}f_{4}\right)$	$\left(f_{4}f_{5}\right)$
node 1	14–17 kHz	17–17.3 kHz	17.5–17.3 kHz
node 2	14–17 kHz	17.7–18 kHz	18.2–18 kHz
node 3	14–17 kHz	18.4–18.7 kHz	18.9–18.7 kHz
node 4	14–17 kHz	19.1–19.4 kHz	19.6–19.4 kHz

**Table 2:** Time parameters of FDM-CSS signal.

Acoustic Node	$\rho_{t_{1}}$	$\rho_{t_{2}}$	$\rho_{t_{3}}$
node 1	30 ms	10 ms	10 ms
node 2	30 ms	10 ms	10 ms
node 3	30 ms	10 ms	10 ms
node 4	30 ms	10 ms	10 ms

**Table 3:** Average measurement error of RDOA under different *LER* values.

Test Point	*LER* = 0.1	*LER* = 0.2	*LER* = 0.3	*LER* = 0.4
1	0.17 m	0.15 m	0.15 m	0.15 m
2	0.24 m	0.24 m	0.24 m	0.24 m
3	0.16 m	0.16 m	0.16 m	0.16 m
4	0.21 m	0.19 m	0.19 m	0.19 m
5	0.45 m	0.23 m	0.23 m	0.23 m
6	0.68 m	0.30 m	0.30 m	0.30 m
7	0.41 m	0.37 m	0.37 m	0.37 m

**Table 4:** Localization results of experiment scene 1.

Region	Evaluation	MF-Max	MF-0.2	MF-0.3	Proposed
Region 1	Max (m)	∞	1.07	2.81	0.75
	Mean (m)	∞	0.29	0.48	0.17
	RMSE(m)	∞	0.40	0.84	0.23
	STD(m)	∞	0.28	0.69	0.15
	92% (m)	∞	0.81	1.31	0.23
	50% (m)	3.89	0.18	0.17	0.14
Region 2	Max (m)	∞	2.08	2.09	0.79
	Mean (m)	∞	0.67	0.89	0.31
	RMSE (m)	∞	0.84	1.09	0.36
	STD (m)	∞	0.50	0.62	0.18
	92% (m)	∞	1.37	2.07	0.56
	50% (m)	2.94	0.63	0.76	0.28

**Table 5:** Localization results of experiment scene 2.

Region	Test Point	MF-Max (m)	MF-0.2 (m)	MF-0.3 (m)	Proposed (m)
Region 1	1	4.94	0.11	0.11	0.15
	2	∞	∞	∞	0.3
	3	2.56	3.33	3.87	1.26
	4	5.80	1.28	1.31	0.25
Region 2	5	2.56	0.21	0.25	0.15
	6	0.31	0.11	0.10	0.11
	7	2.32	0.11	0.11	0.11
	8	3.18	0.15	0.17	0.15
Region 3	9	∞	0.31	0.65	0.15
	10	1.57	0.08	0.09	0.09
	11	2.72	0.07	0.08	0.09
	12	5.28	0.32	0.52	0.15
Region 4	13	6.78	0.09	0.15	0.09
	14	0.64	0.10	0.13	0.08
	15	0.57	0.13	0.42	0.09
	16	0.64	0.18	0.25	0.16
Region 5	17	0.79	0.11	0.11	0.11
	18	0.30	0.21	0.28	0.19
	19	0.86	0.13	0.13	0.10
	20	0.39	0.13	0.14	0.09

## Data Availability

Data are contained within the article.
